# Dopamine Multilocus Genetic Profile, Spontaneous Activity of Left Superior Temporal Gyrus, and Early Therapeutic Effect in Major Depressive Disorder

**DOI:** 10.3389/fpsyt.2020.591407

**Published:** 2020-12-22

**Authors:** Xiaoyun Liu, Zhenghua Hou, Yingying Yin, Chunming Xie, Haisan Zhang, Hongxing Zhang, Zhijun Zhang, Yonggui Yuan

**Affiliations:** ^1^Department of Psychosomatics and Psychiatry, School of Medicine, Zhongda Hospital, Southeast University, Nanjing, China; ^2^Department of Neurology, Zhongda Hospital, School of Medicine, Southeast University, Nanjing, China; ^3^Departments of Clinical Magnetic Resonance Imaging, The Second Affiliated Hospital of Xinxiang Medical University, Xinxiang, China; ^4^Departments of Psychiatry, The Second Affiliated Hospital of Xinxiang Medical University, Xinxiang, China

**Keywords:** major depressive disorder, amplitude of low-frequency fluctuation, multilocus genetic profile scores, dopamine, early therapeutic effect

## Abstract

**Objectives:** This study aimed to examine the interactive effects of dopamine (DA) pathway gene and disease on spontaneous brain activity and further to explore the relationship between spontaneous brain activity and the early antidepressant therapeutic effect in patients with major depressive disorder (MDD).

**Methods:** A total of 104 patients with MDD and 64 healthy controls (HCs) were recruited. The Hamilton Depression Scale-24 (HAMD-24) was used to measure the depression severity. Both groups were given resting-state functional magnetic resonance imaging (rs-fMRI) scan. The amplitude of low-frequency fluctuation (ALFF) was calculated to reflect the spontaneous brain activity based on the rs-fMRI data. After treatment for 2 weeks, depression severity was evaluated again, and HAMD-24 reductive rate was used to measure the therapeutic effect of antidepressants. Multilocus genetic profile scores (MGPS) were used to assess the multi-site cumulative effect of DA pathway gene. The interactive effects of MDD and DA pathway gene on the ALFF of regional brain areas were measured by the multivariate linear regression analysis. Finally, partial correlation analysis (age, sex, education, and illness durations as covariates) was performed to identify the relationship between regional ALFF and therapeutic effect.

**Results:** MDD and DA-MGPS had interactive effects on the left fusiform gyrus (FG_L), right calcarine sulcus (CS_R), left superior temporal gyrus (STG_L), bilateral cerebellum posterior lobe (CPL), bilateral inferior frontal gyrus (IFG), and bilateral superior frontal gyrus (SFG). Partial correlation analysis revealed that the ALFF of STG_L had a significant negative correlation with 2-week HAMD-24 reductive rate (*r* = −0.211, *P* = 0.035).

**Conclusions:** The spontaneous activity of STG_L may be a potential biomarker of antidepressant-related early therapeutic effect underlying the influence of DA pathway genes in MDD.

## Introduction

Major depressive disorder (MDD) is of increasing importance due to its high incidence, recurrence, disability, and burden (1). The overall remission rate of MDD is only about 70% (2). The first-line treatment strategy recommended for MDD is drug treatment, and the most commonly used in a clinical setting are selective serotonin reuptake inhibitors (SSRIs) and selective serotonin-noradrenalin reuptake inhibitors (SNRIs) (3). Besides, these antidepressants are often criticized by the delayed onset of efficacy; usually, it takes 2 weeks or longer on average to work (4, 5). Thus, the biomarkers used to predict the response to a certain antidepressant would help for the selection of clinical medication and the achievement of individualized treatment regimens, this preventing “trial-and-error” in the clinical treatment of MDD and reducing unnecessary waste of medical resources and patients' pains as well as suicide risks. In general, the efficacy assessment window for antidepressants is 4–6 weeks. Nowadays, researchers have suggested to shorten the window to 2 weeks, to evaluate the early therapeutic effect (6), which is more helpful to adjust the treatment strategy in a timely manner.

A genetic component in the etiology of MDD is evident with a 37% heritability (7). The deficits in monoamine system including a reduced neurotransmission of dopamine (DA) are known to be closely related to MDD (8). So far, several DA-related genetic variants have been suggested to modulate endogenous DA neurotransmission, and the most frequently studied are catechol-O-methyltransferase (COMT) gene rs4680, monoamine oxidase A (MAOA) gene rs6323, DA D2 receptor (DRD2) gene rs6277, DRD3 gene rs6280, and DA transporter (DAT) gene rs27072 (8–12).

However, many previous studies of the association of DA and MDD are based on a single polymorphism, which is unlikely to yield significant effects unless very large samples are included (13). Thus, researchers begin to incorporating multiple genes or polymorphisms to determine the pathogenesis of depression from the perspective of genetic pathways.

Genes do not directly encode clinical symptoms, which means there may be some intermediaries between them, whereas imaging genetics is proposed as a bridge linking gene and clinical behavior (7). Gong et al. used the method of imaging genetics and found that the DA multilocus genetic profile played an important role in the reward network and anxious depression in MDD (14). However, previous studies including the studies mentioned above are all the exploration of the relationship between DA pathway genes and depressive symptoms. As far as we know, there is no study on the association between DA pathway genes and the therapeutic effect in MDD.

In this study, using imaging genetics, we aimed to explore the relationship of DA pathway genes on spontaneous brain activity and to determine the brain areas associated with early therapeutic effect in MDD.

## Methods

### Participants

A total of 104 patients with MDD and 64 healthy controls (HC) were included. All participants were right-handed and of Chinese Han ethnicity. All participants signed informed consent forms as approved by the Medical Ethics Committee for Clinical Research of Zhongda Hospital Affiliated with Southeast University. The participants received resting-state functional magnetic resonance imaging (rs-fMRI) scan at baseline. After that, the MDD group received antidepressant treatments.

### Inclusion and Exclusion Criteria

The inclusion criteria for the MDD group were as follows: subjects who (1) were aged ≥18 years; (2) met the criteria for MDD of the Diagnostic and Statistical Manual of Mental Disorders, fourth edition (DSM-IV); (3) had a Hamilton Depression Scale-24 (HAMD-24) score of ≥20. The exclusion criteria were as follows: individuals with a history of (1) other major psychiatric disorders; (2) substance abuse, head trauma, or a loss of consciousness; or (3) cardiac or pulmonary disease that could influence the rs-fMRI scan.

HC subjects were required to have a HAMD-24 score ≤ 7. The exclusion criteria for HC subjects were as follows: (1) a history of neuropsychiatric disease; (2) drug abuse; (3) insobriety; and (4) the presence of implants that could influence the rs-fMRI scan.

### Clinical Evaluations

Depression severity was evaluated by HAMD-24. The therapeutic effect was assessed by the HAMD-24 reductive rate, calculated as: (baseline HAMD-24 score – follow-up HAMD-24 score)/baseline HAMD-24 score × 100%.

### Genetic Data and MGPS Acquisition

DNA was extracted from the subjects' blood using a standard protocol, five polymorphisms that were relatively well studied in the DA pathway (COMT rs4680, MAOA rs6323, DRD2 rs6277, DRD3 rs6280, DAT rs27072) were selected, and genotypes were determined using predesigned Illumina next-generation sequencing and array technologies by Tianhao Biotechnology Company. PLINK 1.9 software was used to calculate the Hardy-Weinberg equilibrium (HWE) tests and linkage disequilibrium statistics (15). MAOA rs6323 deviated from HWE (*P* < 0.05) and the other four SNPs did not (*P* > 0.05), so MAOA rs6323 was excluded from the study. Each genotype of each SNP was scored based on their function in the dopaminergic transmission. Scoring details were provided in Table 1. Then, the scores of four SNPs were added to obtain the multilocus genetic profile score (MGPS) of a subject. The details had been described in previous studies (14, 18).

**Table 1:** The putatively functional single-nucleotide polymorphisms included in the dopamine polygenic score.

**Gene**	**Genotype coding**	**Functional associations**
COMT	rs4680 AA = 1 GA = 0.5 GG = 0	A allele presumably resulted in increased COMT activity (14) and greater synaptic levels of DA (16).
DRD2	rs6277 AA = 1 GA = 0.5 GG = 0	A allele predicted high DRD2 availability in healthy volunteers (17).
DRD3	rs6280 CC = 1 CT = 0.5 TT = 0	C allele was associated with greater reward-related DA release (11) and a higher affinity for DA (9).
DAT	rs27072 TT = 1 CT = 0.5 CC = 0	T allele was associated with lower DAT activity (9).

*COMT, Catechol-O-Methyltransferase; DRD2, D2 Dopamine Receptors; DRD3, D3 Dopamine Receptors; DAT, Dopamine Transporter*.

### rs-fMRI Data Acquisition

rs-fMRI data were obtained using Siemens 3.0-Tesla scanners (Siemens, Erlangen, Germany) at the Second Affiliated Hospital of Xinxiang Medical University and the affiliated Zhongda Hospital of Southeast University. To minimize head motion, the subject's head must be stabilized with a cushion. To reduce scanner noise, every subject was given a pair of earplugs. High-resolution three-dimensional T1-weighted scans were recorded using a magnetization-prepared rapid acquisition with gradient echo (MPRAGE) sequence, and the parameters were repetition time (TR) = 1900 ms, echo time (TE) = 2.48 ms, flip angle (FA) = 9°, acquisition matrix = 256 × 256, field of view (FOV) = 250 × 250 mm^2^, thickness = 1.0 mm, gap = 0, time = 4 min 18 s, and 176 volumes. The parameters of rs-fMRI were TR = 2000 ms, TE = 25 ms, FA = 90°, acquisition matrix = 64 × 64, FOV = 240 × 240 mm^2^, thickness = 3.0 mm, gap = 0 mm, 36 axial slices, time = 8 min, and 240 volumes. During the scans, subjects were instructed to relax with their eyes open and not fall asleep.

### rs-fMRI Data Preprocessing

The rs-fMRI data were preprocessed using the Data Processing Assistant for Resting-State Function (DPARSF 2.3 advanced edition) MRI toolkit (19). The 10 time points that were first scanned were removed to ensure stable longitudinal magnetization and adaptation. The remaining images were processed according to the seven steps: (1) the 36th slice was used as the reference slice according to the number of scanning layers, then temporal differences were corrected, and participants with a head motion >1.5° of angular motion or 1.5 mm of maximum displacement in any direction were ruled out; (2) T1 was co-registered to fMRI and subsequently reoriented; (3) T1-weighted anatomic images were segmented into gray matter, white matter, and cerebrospinal fluid for spatial normalization and then were normalized to the Montreal Neurological Institute space using the transformation parameters estimated with a unified segmentation algorithm (20); the above transformation parameters were applied to the fMRI and the images were resampled with a resolution of 3 × 3 × 3 mm^3^; (4) spatial smoothing was conducted with a 4-mm full-width at half-maximum (FWHM) isotropic Gaussian kernel; (5) the linear trend was removed; (6) the nuisance signals and spiked regressors were regressed out; and (7) a bandpass filter was applied to maintain low-frequency fluctuations (0.01–0.08 Hz).

### ALFF Value Calculation

Based on the preprocessed rs-fMRI data, the ALFF value was calculated by the DPARSF software. First, fast Fourier transformation was used to convert the time series to the frequency domain for each voxel. Then, the power spectrum was obtained. Second, the square root of the power spectrum was calculated and the square root was averaged across a predefined frequency interval. The averaged square root was taken as ALFF, which reflected the absolute intensity of spontaneous brain activity (19). Finally, ALFF was standardized by dividing the whole brain voxel average ALFF to reduce the global effects of variability across subjects.

### Statistical Analyses

One-sample *t* test, independent two-sample *t* test, or chi-squared test was employed to determine the differences in demographic and clinical data using SPSS 20.0. Continuous variables in the statistical results were expressed as the mean ± standard deviation (SD).

We used multivariate linear regression analysis to investigate the potential effects of disease (D) and the DA-MGPS, as well as the interactive effect between D and DA-MGPS (D × MGPS) on spontaneous brain activity using SPM12 (https://www.fil.ion.ucl.ac.uk/spm/download/spm12) after removing the effects of covariates (sex, age, education, and head motion). Results were reported at the significant level of a threshold of two-tailed voxel-wise *P* < 0.01 and cluster level *P* < 0.05 with Gaussian Random Field (GRF) correction. The equation of the multivariate regression analysis was as follows:

$$\begin{array}{l} {\text{m}}_{\text{i}}=\beta 0+\beta 1\times \text{D}+\beta 2\times \text{MGPS}+\beta 3\times \left(\text{D }\times \text{ MGPS}\right)+\beta 4\times \text{Sex}\\ \;+\beta 5\times \text{Edu}+\beta 6\times \text{Age}+\beta 7\times \text{Head Motion}+\;\underset{¸}{\text{o}} \end{array}$$

m_i_ is the Z value of the *i*th voxel across all individuals; β0 is the intercept of the straight line fitting in the model; β1, β2, and β3 are the main effects of disease, MGPS, and the interactive effects of D × MGPS on the ALFF strength of the *i*th voxel on the regional brain areas; in the above linear regression model, sex, education, age, and head motion are discarded as covariates of no interest; and β4, β5, β6, and β7 are the main effects of sex, education, age, and head motion, respectively. The error term o̧ is assumed to have a Gaussian distribution and to be uncorrelated across subjects.

Partial correlation analysis (age, sex, education, and illness durations as covariates) was performed to identify the relationships between regional ALFF and therapeutic effect. The significant level was set at *P* < 0.05.

## Results

### Demographic and Clinical Data

Demographic and clinical data are shown in Table 2. No significant differences were found in sex, age, and education between MDD and HC groups.

**Table 2:** The demographic and clinical data between MDD and HC groups.

**Variable**	**MDD group (*n* = 104)**	**HC group (*n* = 64)**	***P***
Sex (M/F)	50/54	31/33	0.964
Age (years)	44.94 ± 13.28	41.48 ± 13.44	0.105
Education (years)	10.22 ± 4.45	11.84 ± 4.56	0.059
Family history (Y/N)	10/94	–	–
Illness duration (months)	58.36 ± 86.54	–	–
HADM-24 score	31.73 ± 6.07	1.19 ± 1.97	0.000
HAMD-24 reductive rate	0.48 ± 0.16	–	–
MGPS	0.87 ± 0.54	0.74 ± 0.56	0.182

*MDD, Major Depressive Disorder; HC, Healthy Control; HADM-24, Hamilton Depression Scale-24; MGPS, Multilocus Genetic Profile Scores; M/F, Male/Female; Y/N, Yes/No; P, Compare between MDD group and HC group*.

### The Interactive Brain Areas of DA-MGPS and MDD

**Figure 1:**
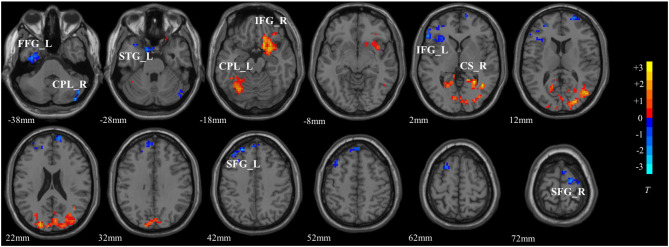
The interactive brain areas of DA pathway gene and major depressive disorder. The colored bar indicated the display window for the threshold *t*-value maps. Hot color represented a higher ALFF value, while blue represented a lower ALFF value. FG_L, left fusiform gyrus; CPL_R, right cerebellum posterior lobe; STG_L, left superior temporal gyrus; CPL_L, left cerebellum posterior lobe; IFG_R, right inferior frontal gyrus; IFG_L, left inferior frontal gyrus; CS_R, right calcarine sulcus; SFG_L, left superior frontal gyrus; SFG_R, right superior frontal gyrus.

The significant interaction effects between diagnosis and DA-MGPS were observed in left fusiform gyrus (FG_L), right calcarine sulcus (CS_R), left superior temporal gyrus (STG_L), bilateral cerebellum posterior lobe (CPL), bilateral inferior frontal gyrus (IFG), and bilateral superior frontal gyrus (SFG). Among these brain areas, CPL_L, IFG_R, and CS_R had an increased ALFF, while others had a decreased ALFF (corrected by GRF; Table 3, Figure 1).

**Table 3:** The interactive brain area of DA-MGPS and MDD.

**Brain regions**	**BA**	**VN**	**Coordinates MNI**			***T* score**	**ES**	***P***
			***X***	***Y***	***Z***			
Left fusiform gyrus	20	97	−36	−9	−39	−3.1844	−0.5090	0.0017
Right cerebellum posterior lobe	–	100	45	−69	−42	−3.7321	−0.5965	0.0003
Left superior temporal gyrus	38	72	−12	6	−27	−2.9737	−0.4753	0.0034
Left cerebellum posterior lobe	–	88	−33	−63	−21	3.4038	0.5440	0.0008
Right inferior frontal gyrus	47	306	15	15	−18	3.8084	0.6087	0.0002
Left inferior frontal gyrus	47	163	−54	30	0	−3.1386	−0.5016	0.0020
Right calcarine sulcus	17	896	27	−51	6	3.829	0.6120	0.0002
Left superior frontal gyrus	9	218	−24	45	42	−3.5127	−0.5614	0.0006
Right superior frontal gyrus	6	117	18	−9	75	−3.2381	−0.5175	0.0015

*BA, Brodmann Area; VN, Voxel Number; ES, Effect Size; MNI, Montreal Neurological Institute*.

The main effects of disease and DA-MGPS on the spontaneous brain activities, as well as the effects of DA-MGPS on spontaneous brain activities in the MDD group and the HC group were presented as Supplementary Materials.

### Correlation Analysis

**Figure 2:**
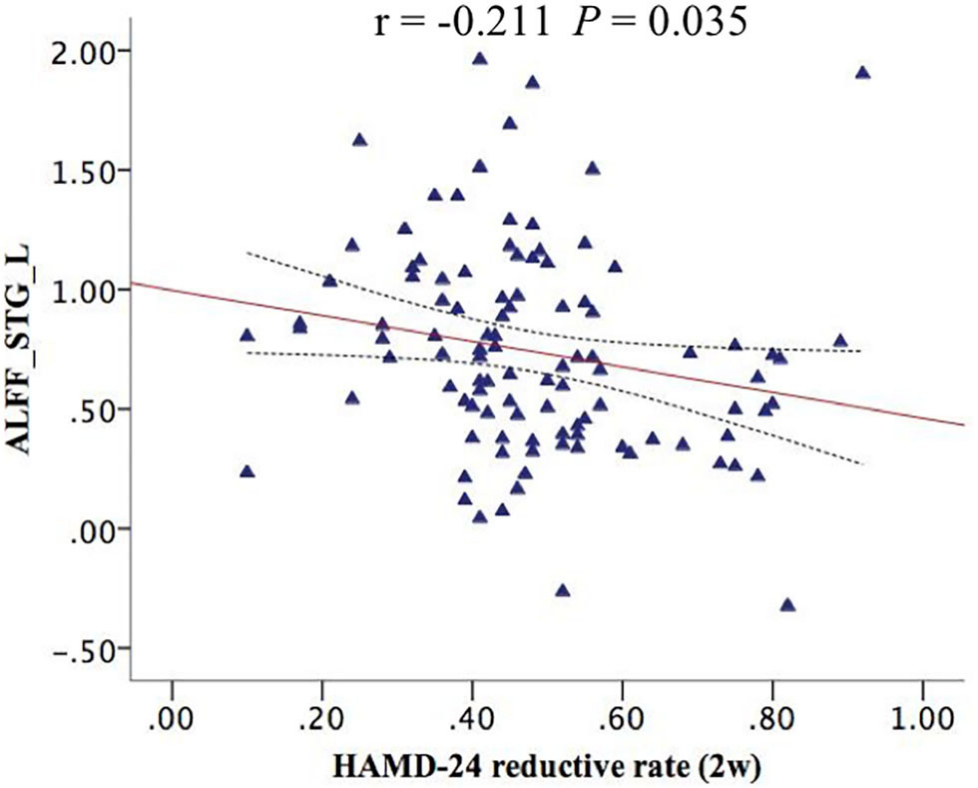
The STG_L ALFF had a negative correlation with 2-week HAMD reductive rate (*r* = −0.211, *P* = 0.035). ALFF_STG_L, the amplitude of low-frequency fluctuation of left superior temporal gyrus.

The correlation analysis between the ALFF value of interactive brain areas with 2-week HAMD reductive rate is shown in Figure 2. We found that the ALFF of STG_L had a significant negative correlation with 2-week HAMD reductive rate (*r* = −0.211, *P* = 0.035; Figure 2).

## Discussion

We found that bilateral CPL, bilateral IFG, bilateral SFG, FG_L, CS_R, and STG_L were both affected by the cumulative effects of DA pathway genes and disease. Zhou et al. found that significant interactive effects of disease × COMT rs4680 were found in the bilateral CS, left SFG, and FG (21). Gong et al. revealed that DA-MGPS was widely associated with the functional connectivity of nucleus accumbens (NAc)-IFG and NAc-STG (14). The above two studies support our findings; however, no studies have found that DA pathway gene and MDD interactively influence cerebellar function. Cerebellum is previously thought to be participated only in motor functions, while increasing evidences demonstrate that this brain area is also involved in emotion processing and cognitive functions (22–24), and the pathophysiology of MDD (25–27). Moreover, a meta-analysis indicated that the posterior and lateral part were mainly responsible for this role (28), which was consistent with ours.

In recent years, the cerebellum has also been proposed as a part of default mode network (DMN). DMN is more active in the resting state, while in the task state, its activities is weakened (29). More and more researches believe that DMN plays an important role in MDD (30, 31), while DMN activities were found to be regulated by DA levels (32). A previous study suggested that DRD2 was critically involved in inhibitory processes, which underlaid the DMN (33). Interestingly, in our study, apart from IFG and FG_L, the other brain areas belonged to DMN, suggesting that the abnormality of DMN in MDD may also be influenced by the DA pathway gene in addition to the disease itself, which of course needs further exploration.

More importantly, we found that the ALFF of STG_L had a negative correlation with a 2-week HAMD-24 reductive rate, indicating that the spontaneous activities of STG_L was closely related with therapeutic effect. The STG contains Heschl's gyrus (primary auditory cortex), planum temporale, and planum polare (auditory association cortical areas), and plays a crucial role in emotional processing, language processing, and auditory memory (34), while the left side of STG is one of the most consistently identified regions involved in the pathophysiology of MDD (35). Zheng et al. assessed the therapeutic effect of 2-week repetitive transcranial magnetic stimulation (rTMS) in MDD and found a decreased functional connection degree (FCD) in right STG; however, the changed FCD is not associated with therapeutic effect (36). Perhaps, the left side of STG is more closely related to the efficacy of MDD rather than the right side.

The negative correlation shows that the lower spontaneous activity in STG_L, the better the early therapeutic effect. Wang et al. also identified a negative correlation between the abnormal function of STG_L and the therapeutic effect in MDD, although they studied the effects of electroconvulsive therapy (ECT), rather than the antidepressants and they measured the functional connectivity density rather than ALFF (37). Meanwhile, they also found a reduced FCD in STG_L compared to the normal controls, which is similar to the decreased ALFF of STG_L underlying the interactive effect of disease × gene in our findings. Combining with the negative correlation, we speculate that the decreased spontaneous activities in STG_L is a compensatory mechanism that contributes to the recovery of MDD. Similarly, Xu et al. suggested that the increase of gray matter volume (GMV) in right STG after a series of ECT reflected the ECT-related brain compensatory mechanisms that contribute to brain structure recovery in MDD (38). Anyway, it is only a conjecture that the abnormal functions or structures of STG are involved in the compensatory mechanisms of MDD, which needs to be further explored in the future.

This study has several limitations. First, our sample size is small, and the control and patient groups are not fully matched; thus, larger and more matched samples are clearly needed to further elucidate. In addition, this is a limited analysis based on a few selected polymorphisms that have been well studied. Further investigation is needed to determine the effects of other DA pathway gene polymorphisms as well. Third, the samples are all Chinese participants, so our results cannot be extrapolated to other ethnic groups. However, samples from the same population are characterized by a high degree of genetic homogeneity, which could be considered both a strength of this work.

## Conclusions

In conclusion, the present study indicates that the spontaneous activity of STG_L may be a potential biomarker of early antidepressants' therapeutic effect underlying the influence of DA pathway genes. This study has the potential to significantly contribute to our understanding of genetic variabilities in the DA system and how DA-related polymorphisms affect the spontaneous brain activities in MDD.

## Data Availability Statement

The datasets presented in this article are not readily available because of the sensitive nature of the questions asked in our study. Requests to access the datasets should be directed to 1360809788@qq.com.

## Ethics Statement

The studies involving human participants were reviewed and approved by Medical Ethics Committee for Clinical Research of Zhongda Hospital Affiliated with Southeast University. The patients/participants provided their written informed consent to participate in this study.

## Author Contributions

XL for data curation and analyses, writing-original draft preparation. ZH, YYi, HaZ, and HoZ for data collection. CX and ZZ for data quality control. YYu for writing-reviewing. All authors contributed significantly and were in agreement with the content of the manuscript.

## Conflict of Interest

The authors declare that the research was conducted in the absence of any commercial or financial relationships that could be construed as a potential conflict of interest.
