# Supplementary material for: Dopamine Multilocus Genetic Profile, Spontaneous Activity of Left Superior Temporal Gyrus, and Early Therapeutic Effect in Major Depressive Disorder
Source: Front Psychiatry. 2020 Dec 22;11:591407. doi: 10.3389/fpsyt.2020.591407 (PMC7782966; doi:10.3389/fpsyt.2020.591407)
Supplement: Supplementary file 1 [file Data_Sheet_1.docx]

**Supplementary Material**

1. **The effect of DA-MGPS in MDD group and HC group.**

The effects of DA-MGPS on spontaneous brain activities in MDD and HC groups had an overlap, namely the right inferior frontal gyrus, which was also the interactive brain area of disease and gene. Meanwhile, there were different brain regions between the two groups, indicating that the cumulative effect of the DA pathway gene had different effects on the spontaneous brain activities in different populations.

**Table 1.** **The effects of DA-MGPS on spontaneous brain activities in MDD**

| Brain regions | BA | Voxel number | Coordinates MNI | | | T-score |
| --- | --- | --- | --- | --- | --- | --- |
|  |  |  | X | Y | Z |  |
| Right inferior frontal gyrus | 47 | 29 | 36 | 42 | 0 | -3.2127 |
| Right medial frontal gyrus | 9 | 22 | 24 | 33 | 24 | -3.913 |

BA: Brodmann Area, MNI: Montreal Neurological Institute.

**Table 2.** **The effects of DA-MGPS on spontaneous brain activities in HC**

| Brain regions | BA | Voxel number | Coordinates MNI | | | T-score |
| --- | --- | --- | --- | --- | --- | --- |
|  |  |  | X | Y | Z |  |
| Right cerebellum posterior lobe | - | 61 | 42 | -81 | -39 | 4.007 |
| Right middle temporal gyrus | 21 | 24 | 42 | -57 | 3 | -3.6147 |
| Left middle temporal gyrus | 21 | 22 | -42 | -60 | 6 | -3.9928 |
| Left claustrum | 13 | 60 | -30 | -24 | 9 | -4.94 |
| Right inferior frontal gyrus | 47 | 21 | 18 | 21 | -12 | -3.9768 |
| Right superior occipital lobe | 18 | 76 | 15 | -87 | 27 | -3.9675 |
| Right calcarine sulcus | 17 | 45 | 27 | -54 | 6 | -4.3408 |
| Left corpus callosum | 29 | 218 | -24 | 45 | 42 | -3.5127 |
| Right superior frontal gyrus | 10 | 35 | 21 | 60 | 12 | 4.0261 |

BA: Brodmann Area, MNI: Montreal Neurological Institute.

1. **The** **main effect of D and main effect of DA-MGPS**

The main effects of disease on the spontaneous brain activities were distributed in right cerebellum posterior lobe, left cerebellum posterior lobe, left anterior cingulate cortex, left calcarine sulcus, and right paracentral lobe (Table 3). And that of DA-MGPS were right cerebellum posterior lobe, left cerebellum posterior lobe, right inferior frontal gyrus, right superior occipital gyrus, right middle temporal gyrus, right calcarine sulcus, and right cuneus (Table 4).

**Table 3.** **T****he main effects of disease on the spontaneous brain activities**

| Brain regions | BA | Voxel number | Coordinates MNI | | | T-score |
| --- | --- | --- | --- | --- | --- | --- |
|  |  |  | X | Y | Z |  |
| Right cerebellum posterior lobe | - | 244 | 42 | -72 | -24 | -5.1569 |
| Left cerebellum posterior lobe | - | 145 | -18 | -87 | -24 | -4.6779 |
| Left anterior cingulate cortex | 24 | 2838 | -12 | 27 | -6 | 5.5602 |
| Left calcarine sulcus | 17 | 135 | -21 | -72 | 12 | 3.5994 |
| Right paracentral lobe | 6 | 778 | 3 | -21 | 75 | -5.4241 |

BA: Brodmann Area, MNI: Montreal Neurological Institute.

**Table 4. The main effects of** **DA-MGPS on the spontaneous brain activities**

| Brain regions | BA | Voxel number | Coordinates MNI | | | T-score |
| --- | --- | --- | --- | --- | --- | --- |
|  |  |  | X | Y | Z |  |
| Right cerebellum posterior lobe | - | 27 | 45 | -69 | -42 | -3.7321 |
| Left cerebellum posterior lobe | - | 21 | -33 | -63 | -21 | 3.4038 |
| Right inferior frontal gyrus | 47 | 98 | 15 | 15 | -18 | 3.8084 |
| Right superior occipital gyrus | 19 | 55 | 24 | -87 | 12 | 3.3632 |
| Right middle temporal gyrus | 21 | 24 | 45 | -72 | 12 | 3.4569 |
| Right calcarine sulcus | 17 | 24 | 27 | -51 | 6 | 3.829 |
| Right cuneus | 18 | 53 | 3 | -78 | 18 | 3.5155 |

BA: Brodmann Area, MNI: Montreal Neurological Institute.
